# Phylogenetic classification of *Escherichia coli *O157:H7 strains of human and bovine origin using a novel set of nucleotide polymorphisms

**DOI:** 10.1186/gb-2009-10-5-r56

**Published:** 2009-05-22

**Authors:** Michael L Clawson, James E Keen, Timothy PL Smith, Lisa M Durso, Tara G McDaneld, Robert E Mandrell, Margaret A Davis, James L Bono

**Affiliations:** 1 United States Department of Agriculture (USDA), Agricultural Research Service (ARS), US Meat Animal Research Center (USMARC), State Spur 18D, Clay Center, NE 68933, USA; 2 USDA, ARS, Western Regional Research Center, Buchanan St, Albany, CA 94710, USA; 3 Washington State University, Department of Pathology, Bustad Hall, Pullman, WA 99164-7040, USA; 4 Current address: University of Nebraska, Great Plains Veterinary Educational Center, Clay Center, NE 68933, USA

## Abstract

**Background:**

Cattle are a reservoir of Shiga toxin-producing *Escherichia coli *O157:H7 (STEC O157), and are known to harbor subtypes not typically found in clinically ill humans. Consequently, nucleotide polymorphisms previously discovered via strains originating from human outbreaks may be restricted in their ability to distinguish STEC O157 genetic subtypes present in cattle. The objectives of this study were firstly to identify nucleotide polymorphisms in a diverse sampling of human and bovine STEC O157 strains, secondly to classify strains of either bovine or human origin by polymorphism-derived genotypes, and finally to compare the genotype diversity with pulsed-field gel electrophoresis (PFGE), a method currently used for assessing STEC O157 diversity.

**Results:**

High-throughput 454 sequencing of pooled STEC O157 strain DNAs from human clinical cases (n = 91) and cattle (n = 102) identified 16,218 putative polymorphisms. From those, 178 were selected primarily within genomic regions conserved across *E. coli *serotypes and genotyped in 261 STEC O157 strains. Forty-two unique genotypes were observed that are tagged by a minimal set of 32 polymorphisms. Phylogenetic trees of the genotypes are divided into clades that represent strains of cattle origin, or cattle and human origin. Although PFGE diversity surpassed genotype diversity overall, ten PFGE patterns each occurred with multiple strains having different genotypes.

**Conclusions:**

Deep sequencing of pooled STEC O157 DNAs proved highly effective in polymorphism discovery. A polymorphism set has been identified that characterizes genetic diversity within STEC O157 strains of bovine origin, and a subset observed in human strains. The set may complement current techniques used to classify strains implicated in disease outbreaks.

## Abstract 2

Novel SNPs from human and bovine O157:H7 E. coli isolates are mapped, revealing that the majority of human disease is caused by a bovine subset of this strain.

## Background

Shiga toxin-producing *Escherichia coli *O157:H7 (STEC O157) recently emerged as a cause of diarrhea, hemorrhagic colitis, and hemolytic uremic syndrome (HUS) [1]. STEC O157 cause an estimated 73,480 illnesses each year in the United States [2] and probably evolved from a progenitor of *E. coli *O55:H7, a source of infantile diarrhea [3]. The STEC O157 5.5-Mb genome contains a 4.1-Mb backbone that is shared with *E. coli *K-12 and thought to be conserved across most *E. coli *serotypes [4,5]. Much of the remaining genome originates from horizontal transfer, with a significant contribution from bacteriophages [4,5]. The loss and gain of genes through horizontal transfer, coupled with nucleotide variation distributed throughout the STEC O157 genome, serve in both recording the evolution and defining the diversity of this pathogenic serotype [6-8].

Detection of genetic diversity between STEC O157 strains is an important component of outbreak investigations. Heterogeneity between STEC O157 strains has been detected through multilocus sequence tagging [9], octamer and PCR-based genome scanning [10,11], phage typing [12,13], multiple-locus variable-number tandem repeat analysis [14], microarrays [15,16], nucleotide polymorphism assays [8], phage integration patterns coupled with genome polymorphisms [17,18], and pulsed-field gel electrophoresis (PFGE) [19,20]. Of these, PFGE is currently the method of choice for distinguishing between STEC O157 strains implicated in outbreaks [21], and entails standardized chromosome digestions with *Xba*I, and separation of DNA segments through gel electrophoresis[22]. Differing banding patterns are used to identify genetic diversity between STEC O157 strains. However, PFGE does not effectively show evolutionary descent between epidemiologically related strains [8,23]. Additionally, the standardized PFGE method is unreliable for determining genetic relatedness between STEC O157 strains that are epidemiologically unrelated [24].

Nucleotide polymorphisms, if sufficiently present within microbial populations, such as STEC O157, are highly amenable for determining genetic relatedness and descent between either epidemiologically related or unrelated strains [25]. Single nucleotide polymorphisms (SNPs) have been recently identified throughout the STEC O157 genome [15,16,26] and some have been used to identify variation between STEC O157 strains originating from clinically ill humans [8]. Thirty-nine SNP-based STEC O157 genotypes were identified that defined nine phylogenetic clades, of which one associated with increased hemolytic uremic syndrome, a serious complication of STEC O157 infection [8]. Thus, SNPs have been employed in the classification of STEC O157 by phylotype, and for distinguishing a subpopulation with increased human virulence.

Cattle are a reservoir of STEC O157 and harbor subtypes that are not typically observed in humans [10,26]. Consequently, SNPs ascertained exclusively with strains associated with human outbreaks [16] may be ineffective at distinguishing a greater proportion of STEC O157 genetic diversity present in cattle. Given that strains of any genetic subtype may be drawn into a human outbreak investigation, and/or food recall, an ability to detect the fullest spectrum of STEC O157 genetic diversity with nucleotide polymorphisms would be useful in any STEC O157 investigation. Additionally, a greater understanding of STEC O157 genetic diversity, and how it relates to human pathogenesis, may lead to the identification of alleles that are directly involved with increased human virulence.

The main goal of this study was to sequence the genomes (1×) of 193 diverse STEC O157 strains and identify a set of nucleotide polymorphisms that classify STEC O157 of either bovine or human origin by genotype. Reported here are 42 unique polymorphism-derived STEC O157 genotypes that are tagged by a minimal set of 32 polymorphisms. Phylogenetic trees produced by the genotypes are split into clades that represent strains of cattle origin, or cattle and human origin. These results indicate that heterologous members of the STEC O157 serotype are distinguishable through nucleotide polymorphisms, and support the notion that a subset of STEC O157 harbored in cattle causes the majority of human disease.

## Results

### Sequencing coverage of 193 STEC O157 strains

Approximately 1× genome coverage of 193 STEC O157 strains was obtained through 454 GS FLX shotgun sequencing of three STEC O157 DNA pools (see Additional data file 1 for supplementary strain, PFGE, and genotype information). The DNA pools were designed to account for: host origin; and the alleles of a polymorphism in the translocated intimin receptor gene (*tir *255T>A), as STEC O157 with the *tir *255T>A A allele are rarely isolated from clinically ill humans [26]. A total of 1.306 Gb of genomic sequence was obtained from DNA pools of: 51 strains of bovine origin, *tir *255 T>A A allele (346.2 Mb); 51 strains of bovine origin *tir *255T>A T allele (402.6 Mb); and 91 strains of human origin, of which all had the *tir *255T>A T allele (557.5 Mb). Given that the STEC O157 genome is approximately 5.5 Mb, the depth of sequence obtained for each of the three pools averages to slightly more than 1× whole genome coverage for each of the 193 strains sequenced in this study.

### Polymorphism identification and validation

**Figure 1:**
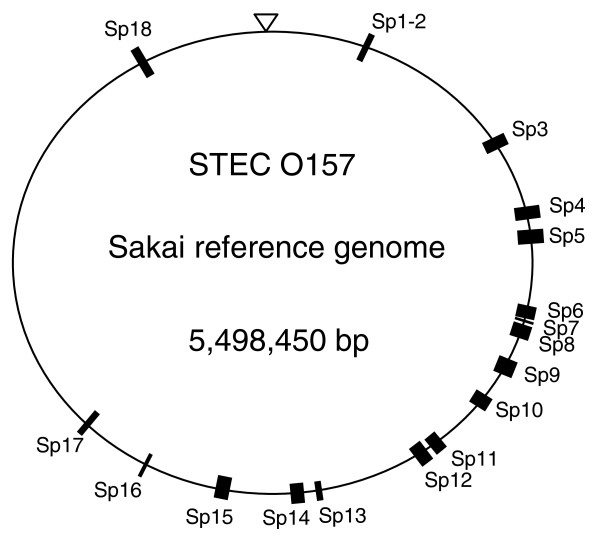
Regions of the STEC O157 genome (Sakai reference strain) targeted for polymorphism validation. Black rectangles represent known phage or phage remnant integrations (Sakai prophages; Sp1-18, [4]) that were not queried for polymorphism validation. All other regions, totaling 87.8% of the genome, were included for polymorphism validation. The triangle points to nucleotide 1 of the Sakai genome sequence [GenBank:NC_002695].

A total of 16,218 putative nucleotide and/or insertion deletion polymorphisms were identified and mapped onto the Sakai STEC O157 reference genome with Roche GS Reference Mapper Software (Nutley, NJ, USA). Of these, 9,528 mapped to prophages integrated throughout 12.2% of the Sakai genome (Figure 1). Phage integration loci are problematical for both SNP discovery and validation, as multiple integrations within the STEC O157 genome have resulted in large stretches of paralogous sequence that are virtually indistinguishable from one another. As a result, apparent polymorphisms may actually be differences between two or more highly similar sites in the genome rather than representing true variation at a single nucleotide locus. In addition, assay design in these highly repetitive sequences is impractical. Consequently, putative polymorphisms identified within these sites were not queried with validation assays in this study. Of the remaining 6,690 putative polymorphisms, 1,735 were identified via the STEC O157 DNA pool of human strains, and 4,955 were identified via the DNA pools of cattle strains.

Matrix-assisted laser desorption-ionization time-of-flight (MALDI-TOF) genotype validation assays were developed for 227 putative polymorphisms based on their minor allele frequencies in one or more of the STEC O157 DNA pools. The minor alleles of 169 polymorphisms were observed exclusively in one of the three DNA pools at a frequency of 15% or higher (human strain DNA pool (n = 18), bovine strain DNA pool, *tir *255T>A T allele (n = 81), bovine strain DNA pool, *tir *255T>A A allele (n = 70)). Additionally, 58 polymorphisms were included where the minor allele was observed in both bovine DNA pools with a minor allele frequency of 10% or higher in one or both pools. MALDI-TOF genotyping of the 227 polymorphisms across 261 STEC O157 strains (Additional data file 1) indicated that 21 putative polymorphisms resided in duplicated genomic regions as a subset of STEC O157 strains yielded heterozygous genotypes. Another 28 putative polymorphisms proved either intractable for genotyping or yielded monomorphic genotypes. Of 178 polymorphisms validated by MALDI-TOF genotyping, 139 reside in open reading frames with 86 predicted non-synonymous or premature stop codon allele variants. Additionally, 154 reside on the conserved genomic backbone of *E. coli *(see Additional data file 2 for supplementary polymorphism information).

### Identification of polymorphism-derived genotypes in STEC O157 strains of human and cattle origin

**Figure 2:**
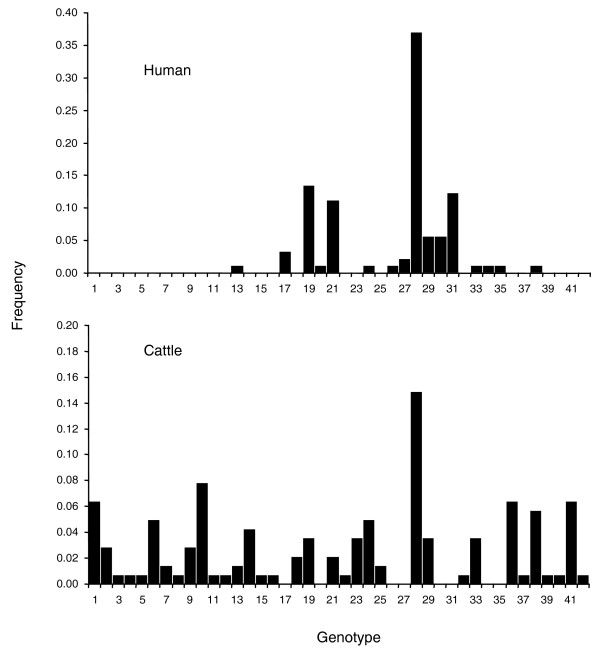
Frequencies of 42 polymorphism-derived genotypes in STEC O157 strains of human and cattle origin.

Concatenation of 178 polymorphism alleles for each of the STEC O157 strains genotyped in this study yielded 42 unique polymorphism-derived genotypes that are delineable with a minimal subset of 32 'tagging' polymorphisms (see Additional data files 3 and 4 for polymorphism-derived genotypes based on all 178 and the 32 tagging polymorphisms, respectively). A total of 34 of the polymorphism-derived genotypes were observed in STEC O157 strains of cattle origin, 16 were observed in strains of human origin, with 8 observed in strains of both human and cattle origin (Figure 2; Additional data file 1). Eight genotypes were observed exclusively in strains of human origin and 26 were observed exclusively in strains of bovine origin. Of particular interest, the same STEC O157 genotype (genotype 28) had the highest overall frequency in STEC O157 strains of human and bovine origin (Figure 2), indicating that STEC O157 of this genetic background may have an advantage in populating cattle and/or causing disease in humans.

### Phylogenetic analyses of STEC O157 polymorphism-derived genotypes

**Figure 3:**
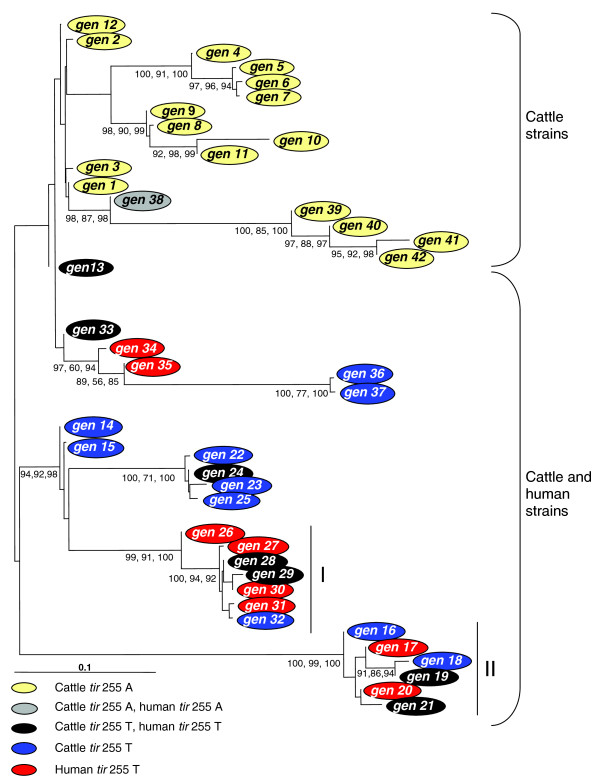
Neighbor-joining tree of full-length polymorphism-derived genotypes. The triplicate sets of numbers on the tree represent bootstrap values from neighbor-joining, parsimony, and maximum-likelihood algorithms, respectively. Outer taxonomic unit genotype numbers correspond with genotype sequences recorded in Additional data file 3. The outer taxonomic units are color coded by genotype for the *tir *255 T>A polymorphism and host origin. Roman numerals depict two subclades that account for 92% of the human STEC O157 strains genotyped in this study. The scale bar represents substitutions per site.

Neighbor-joining, parsimony, and maximum-likelihood trees were generated for the 42 polymorphism-derived genotypes using 178 polymorphism alleles, and the minimal set of 32 tagging polymorphism alleles. Both allele data sets yielded similar trees; however, bootstrap values were lower overall in trees generated with the minimal set of 32 tagging polymorphism alleles, as this set contained a reduced amount of phylogenetic information (Figure 3; see Additional data file 5 for a phylogenetic tree based on the 32 tagging polymorphism alleles). The trees were used to depict the genetic relatedness of STEC O157 strains of known host origin and *tir *255T>A allele status. The neighbor-joining tree in Figure 3 was constructed from 178 polymorphism alleles, and shows a monophyletic cluster of all 17 polymorphism-derived genotypes with the *tir *255T>A A allele. Strains from 66 cattle originating from the US, Japan, Scotland, and Australia had the *tir *255T>A A allele and one of the 17 polymorphism-derived genotypes. Additionally, the one STEC O157 strain of human origin included in this study that had the *tir *255T>A A allele also had a genotype contained within the cluster (Additional data files 1, 3 and 4). The remaining 25 polymorphism-derived genotypes represent STEC O157 strains isolated from humans or cattle that all have the *tir *255T>A T allele. These genotypes cluster together in subclades that are strongly supported by neighbor-joining, parsimony, and maximum-likelihood algorithms (Figure 3). Ninety-two percent of the human STEC O157 strains genotyped in this study placed within two subclades on the tree (Figure 3).

**Figure 4:**
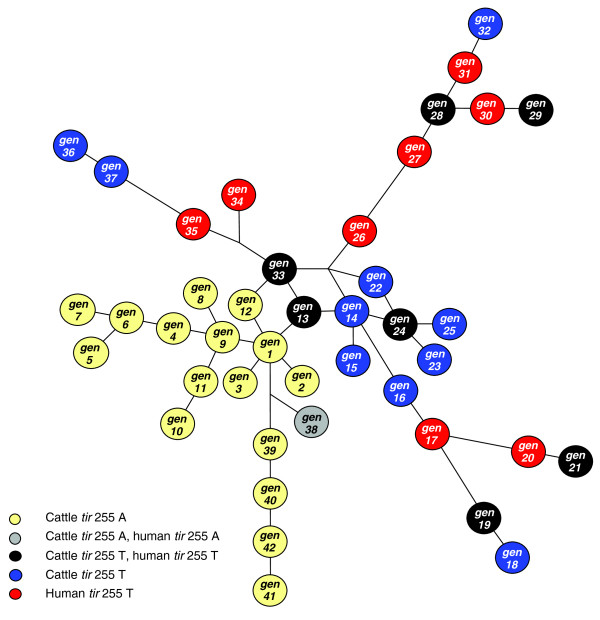
Median-joining network of polymorphism-derived genotypes tagged with a minimal set of 32 polymorphisms. Taxonomic unit genotype numbers correspond with genotype sequences recorded in Additional data file 4 and the units are color coded by genotype for the *tir *255 T>A polymorphism and host origin.

To determine the extent to which the polymorphism-derived genotypes can be used to distinguish STEC O157 genetic relatedness, a median-joining network was constructed from the 32 tagging polymorphism data set (Figure 4). Unlike the neighbor-joining, parsimony, and maximum-likelihood trees, which placed genotypes exclusively as outer taxonomic units, the median-joining network allowed for genotypes to be placed as either internal or outer nodes of the network. Nodes on linear, open, connecting lines on this network represent stepwise evolutionary descent. Nodes on circular, closed loops in the center of the network indicate that either convergent evolution or recombination occurred within some STEC O157 strains (Figure 4) [8]. Given that lateral-gene transfer is a fundamental component of STEC O157 biology and pathogenesis, and that a tri-allelic polymorphism was identified in this study (Additional data file 2; nucleotide position 3,506,470, Additional data files 3 and 4), either scenario is likely and both confound interpretations of genetic relatedness on the network, which assumes stepwise evolutionary descent. Consequently, the loops within the center of the network provide a natural barrier in determining genetic relatedness between STEC O157 genotypes (Figure 4).

### Comparison of PFGE and polymorphism-derived genotype diversity

**Figure 5:**
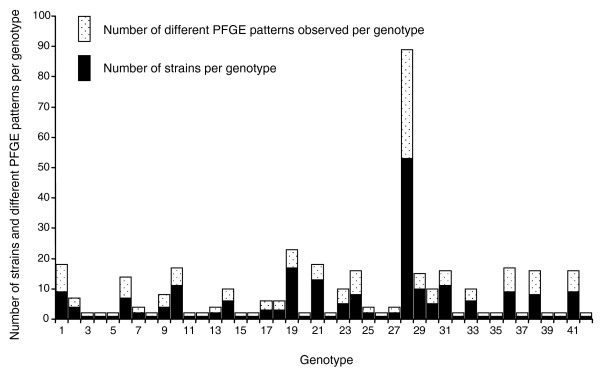
Number of strains and PFGE patterns per genotype. Each stacked bar represents a total of the number of strains per genotype and the number of different PFGE patterns observed per genotype. The black portion of the bars represents the number of strains per genotype. The white speckled portions of the bars represent the number of different PFGE patterns observed per genotype.

**Figure 6:**
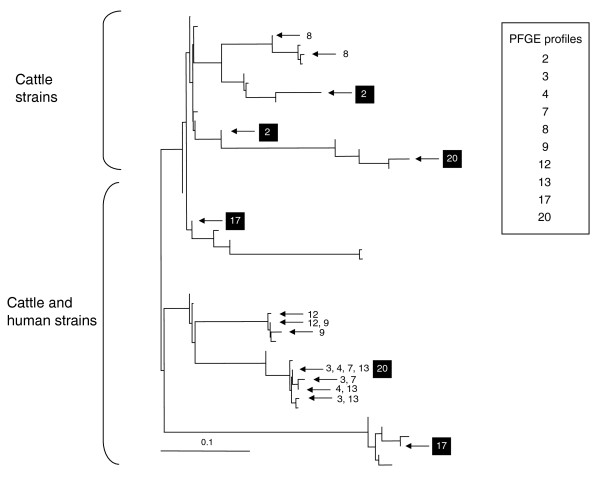
Neighbor-joining tree placement of ten PFGE profiles onto corresponding polymorphism-derived genotypes. Each of the ten profiles was observed with more than one STEC O157 strain. The PFGE profile numbers match with those in Additional data file 1. Identical PFGE profiles that occurred with distantly related STEC O157 strains as determined with polymorphism-derived genotypes are highlighted in black.

PFGE patterns and polymorphism-derived genotypes were compared between 227 epidemiologically unrelated STEC O157 strains using unambiguous PFGE patterns and polymorphism derived-genotypes (Additional data file 1). A conservative standard was employed for distinguishing differing PFGE patterns, where only identical banding patterns were assigned to the same PFGE group. We observed 154 PFGE patterns and 42 polymorphism-derived genotypes between the strains, with multiple PFGE patterns observed on 24 genotypes (Figure 5; Additional data file 1). A total of 131 PFGE patterns and 18 polymorphism-derived genotypes manifested as singletons in this study (Additional data file 1). Of 23 PFGE patterns observed in more than one strain, 10 occurred with strains having different polymorphism-derived genotypes, with 3 PFGE patterns each manifesting in strains of markedly different genetic backgrounds (Figure 6). This result indicates that the polymorphism-derived genotypes described in this study have an immediate utility in distinguishing genetically distinct STEC O157 strains that appear identical by PFGE profile.

## Discussion

GS FLX sequences are clonal in origin because they are ultimately derived from a single strand of DNA. Consequently, high- and low-frequency polymorphism alleles can be detected through GS FLX sequencing of pooled DNA libraries. We took advantage of this attribute by designing STEC O157 DNA pools that were sorted by host origin phenotype (cattle or human), and genotype for the *tir *255 T>A polymorphism. Because the *tir *255 T>A A allele is rarely observed in STEC O157 isolated from humans, STEC O157 DNAs of cattle origin could be separated into two pools, one representing a portion of STEC O157 diversity that appears primarily in cattle, and one representing a portion of STEC O157 diversity that may or may not appear in clinically ill humans. These two pools were complemented with the DNA pool of STEC O157 strains isolated from clinically ill humans. By selecting polymorphisms where the minor allele was observed at a relatively high frequency in either the STEC O157 DNA pool of human strains or at least one of the two cattle strain DNA pools, 42 polymorphism genotypes were identified that cover a large spectrum of STEC O157 diversity present in cattle, and a subset of genotypes that manifests in clinically ill humans.

While this study defined a sub-lineage of STEC O157 that is poorly represented in humans, a genetic mechanism for causing this host restriction is unknown. The *tir *255T>A polymorphism is a likely candidate as the translocated intimin receptor protein is part of the STEC O157 type-three secretion system and facilitates bacterium attachment to enterocyte cells within the colon and subsequent effacement [27]. Additionally, the T>A substitution encodes a non-synonymous replacement of aspartate for glutamate in the translocated intimin receptor protein. However, the *tir *255T>A polymorphism is not known to directly affect STEC O157 virulence in humans [26], and this study identified 1,735 putative polymorphisms (outside of phage integration sites) with minor alleles exclusively observed in the human STEC O157 DNA pool. This finding complicates interpretations regarding a *tir *255T>A polymorphism effect on human virulence. Regardless of knowing which alleles directly impact the ability of STEC O157 to cause human disease, an ability to track and identify variation linked with the *tir *255T>A A allele is important, as one human strain in this study had the *tir *255T>A A allele and a polymorphism-derived genotype that fell within the monophyletic clade typically found in cattle.

A majority of STEC O157-induced human disease sampled in this study was caused by strains that have followed one of two overarching lines of descent, as 92% of all human strain polymorphism-derived genotypes were placed within two large subclades (Figures 3 and 4; Additional data file 5). These two clades are separated from one another by both orthologous descent and probable recombination (Figure 4) and may be split out further into smaller clade sets [8]. It is likely that variation between the subclades, or variation among genotypes within a subclade, may associate with human virulence, as this has been previously demonstrated with the US spinach outbreak strain of 2006 [8], which had a higher rate of hospitalization and hemolytic uremic syndrome than other outbreak strains [28]. The US spinach outbreak strain was included in this study and has a polymorphism-derived genotype (genotype 21) that differs from all others, in that only it contains the minor alleles of two non-synonymous polymorphisms (Additional data files 2 and 3, N-acetylglutamate synthase: alanine to serine (position 3,672,410), and cytochrome c nitrite reductase: arginine to histidine (position 5,141,169)). These polymorphisms were not characterized in a previous study of STEC O157 polymorphism-derived genotypes and human virulence [8] as only four polymorphisms used in that study coincide with the 178 described here.

The polymorphisms validated in this study primarily reside in the conserved backbone of *E. coli *and some may be informative across *Escherichia *species. PFGE, the current gold standard for assessing STEC O157 genetic diversity [21], primarily detects insertions and/or deletions within genomic regions specific to STEC O157 [29]. Consequently, PFGE and the polymorphism-derived genotypes described in this study target different regions of the STEC O157 genome that do not share a common phylogeny. It is not surprising that PFGE diversity surpassed polymorphism-derived genotype diversity overall, given that PFGE patterns are known to change between subcultures of the same strain of STEC O157:H7 [30] and that plasmid migration within PFGE can be unpredictable [23]. Future studies should be conducted that compare STEC O157 diversity assessed with the polymorphism-derived genotypes and PFGE using outbreak samples. However, given that ten different PFGE patterns were each observed in two or more strains with different polymorphism genotypes, the 42 polymorphism-derived genotypes identified in this study have immediate potential to resolve genetically distinct STEC O157 strains comprising an outbreak investigation that may be indistinguishable by PFGE.

## Conclusions

The method of pooling large numbers of phenotyped STEC O157 strain DNAs and subsequent high throughput 454 sequencing proved extremely efficient for the identification of variation within and between pooled populations, and resulted in the identification of 178 polymorphisms that collectively define 42 unique STEC O157 genotypes. The genotypes characterize genetic diversity and relatedness within STEC O157 strains of bovine origin, and a subset observed in human strains. We identified a minimal set of 32 polymorphisms that tag all 42 genotypes, and show that this set can detect genetically diverse STEC O157 strains that are indistinguishable by PFGE.

## Materials and methods

### Bacterial strains

STEC O157 strains of bovine origin (n = 102) that varied by source, and epidemiologically unrelated human clinical STEC O157 strains (n = 91) were used for polymorphism discovery (Additional data file 1) [4,31-37]. Each strain was characterized as STEC O157 by an enzyme-linked immunosorbent assay using an O157 monoclonal antibody and multiplex PCR for *stx1*, *stx2*, *eae*, *hlyA*, *rfb*_*O*157 _and *fliC*_*H*7 _[38-41]. Additionally, each strain was genotyped for a polymorphism residing within the translocated intimin receptor gene (*tir *255 T>A) [26]. A total of 261 STEC O157 strains, 164 isolated from cattle and 97 isolated from human were targeted for genotyping of: *tir *255T>A; 178 polymorphisms identified in this study; and PFGE (Additional data file 1).

### DNA isolation

Genomic DNA was extracted from STEC O157 strains using Qiagen Genomic-tip 100/G columns (Valencia, CA, USA) and a modified manufacturer's protocol. Following overnight growth in 5 ml of Luria broth, bacteria were pelleted by centrifugation at 5,000 × g for 15 minutes, re-suspended in Qiagen buffer B1 containing RNase A (0.2 mg/ml), and vortexed per the manufacturer's instructions. Importantly, the samples were then incubated at 70°C for 10 minutes, vortexed, and equilibrated at 37°C (failure to include the 70°C step frequently resulted in the columns becoming plugged and/or a significant decrease in DNA yield). Following the addition of 80 μl lysozyme (100 mg/ml), 100 μl proteinase K (Qiagen), and a 37°C incubation for 30 minutes, the DNAs were extracted and air dried per the manufacturer's protocol. Purified DNAs were suspended in 500 μl TE (10 mM Tris pH 8.0, 0.1 mM EDTA) and incubated for 2 hours at 50°C, followed by an overnight incubation at room temperature with gentle mixing. Strain DNA preparations were assessed by 260 nm/280 nm absorptions, which were determined with a NanoDrop Technologies ND-1000 spectrophotometer (Wilmington, DE, USA), and by gel electrophoresis.

### STEC O157 DNA pools, GS FLX sequencing, and polymorphism identification

Three STEC O157 DNA pools were created for GS FLX sequencing and polymorphism discovery. One consisted of DNAs from 51 STEC O157 strains (3 μg/strain), all of cattle origin and all with the *tir *255 T>A A allele. Another consisted of DNAs from 51 STEC O157 strains (3 μg/strain), all of cattle origin and all with the *tir *255 T>A T allele. Another consisted of DNAs from 91 STEC O157 strains (3 μg/strain) originating from clinically ill humans, all with the *tir *255 T>A T allele. Genomic libraries were prepared from each of the three DNA pools for Roche 454 GS FLX shot-gun sequencing according to the manufacturer's protocol (Nutley, NJ, USA). A total of 11 emulsion-based PCRs and sequencing runs were performed, three for the DNA pool of cattle origin, *tir *255T>A A allele, three for the DNA pool of cattle origin, *tir *255T>A T allele, and five for the DNA pool of human origin. SNPs were mapped to a reference sequence of STEC O157 (Sakai strain) and identified with Roche GS Reference Mapper Software (version 1.1.03).

### Polymorphism genotyping

A file containing all targeted polymorphisms was prepared for assay design and multiplexing by MassARRAY^® ^assay design software as recommended by the manufacturer (Sequenom, Inc., San Diego, CA, USA). A target of maximum 36 and minimum 21 polymorphisms per multiplex was set for design, with default settings for all other parameters. Seven multiplexes containing 225 polymorphisms were designed (average 32 polymorphisms per multiplex, range 21 to 36). Assays were performed using iPLEX Gold^® ^chemistry on a MassARRAY^® ^genotyping system as recommended by the manufacturer (Sequenom Inc.). Genotypes designated as high confidence by the Genotyper^® ^software were accepted as correct; those with lower confidence (marked 'aggressive' in the software) were manually inspected. Replicate iPLEX assays and/or Sanger sequencing were used to verify genotypes.

### Polymorphism-derived genotype analyses

The alleles of 178 polymorphisms were concatenated by physical order along the STEC O157 genome for 261 STEC O157 strains and aligned using Clustal X (version 1.83) [42]. Redundant polymorphism-derived genotypes were identified using TreePuzzle (version 5.2) [43,44], and removed from Clustal X alignments. Neighbor-joining and parsimony phylogenetic trees were generated using a collection of software programs in PHYLIP (version 3.65, Consense, DnaDist, DnaPars, Neighbor, Retree, Seqboot) [45]. To construct a neighbor-joining tree, a distance matrix was first produced in DnaDist using an F84 distance model of substitution and a transition/transversion ratio of 2. The output of DnaDist was used to construct a neighbor-joining tree in Neighbor, which was mid-point rooted using Retree. Neighbor-joining bootstraps (1,000) were determined with Seqboot, DnaDist, Neighbor, and Consense. A parsimony tree with 1,000 bootstraps was generated with Seqboot, DnaPars (best tree thorough search) and Consense. Maximum-likelihood trees were generated in Tree-Puzzle (version 5.2) with 10,000 puzzling steps and an HKY model of substitution. Neighbor-joining, parsimony, and maximum-likelihood trees were all viewed in TreeView (version 1.6.6) [46].

Haploview v 4.1 [47] was used to identify a minimal set of polymorphisms (tagging polymorphisms) that distinguish each of the unique polymorphism-derived genotypes observed in this study. All 178 polymorphism genotypes were used to infer STEC O157 haplotypes in Haploview at a haplotype frequency threshold of 0% or higher. Neighbor-joining, parsimony, and maximum-likelihood trees were generated from concatenated tagging polymorphism genotypes using model assumptions identical to those used for the full genotype data sets. Additionally, a median-joining network was constructed in Network (version 4.5.0.2) [48] for the concatenated tagging polymorphism genotypes.

### Pulsed field gel electrophoresis

The standardized PFGE method [49] was performed on 261 STEC O157 strains that were also targeted for SNP genotyping (Additional data file 1). Gel images were analyzed using Bionumerics (Applied Maths, Sint-Martens-Latem, Belgium), and banding patterns were clustered using an unweighted pair-group method with arithmetic mean algorithm and a band-based Dice coefficient. Default tolerance settings were used. No restriction enzymes additional to *Xba*I were used. Strains were assigned to the same PFGE group only if *Xba*I banding patterns were indistinguishable.

## Abbreviations

MALDI-TOF: matrix-assisted laser desorption-ionization time-of-flight; PFGE: pulsed-field gel electrophoresis; SNP: single nucleotide polymorphism; STEC O157: Shiga toxin-containing *Escherichia coli *O157:H7.

## Authors' contributions

MLC conducted experimental design and data generation, analyzed 454 GS FLX and MALDI-TOF results, performed phylogenetic analyses, and wrote the manuscript. JEK conceived the project, characterized STEC O157 strains, and participated in 454 GS FLX sequencing design. TPLS participated in experimental design and STEC O157 DNA purification, conducted 454 GS FLX library construction and sequencing, and MALDI-TOF genotyping. LMD characterized STEC O157 strains, performed and analyzed PFGE, and participated in STEC O157 DNA purification. TGM participated in STEC O157 DNA purification and 454 GS FLX library construction and sequencing. REM characterized epidemiologically related STEC O157 strains. MAD characterized an international collection of STEC O157 strains and provided PFGE results. JLB participated in experimental design, conducted STEC O157 characterizations, culture, and DNA isolations, and analyzed 454 GS FLX and MALDI-TOF results.

## Additional data files

The following additional data are available with the online version of this paper: a table of STEC O157 strains used in this study with their corresponding PFGE patterns and polymorphism-derived genotypes (Additional data file 1); a table of nucleotide polymorphism allele frequencies in STEC O157 strains of bovine and human origin (Additional data file 2); a table of STEC O157 genotypes defined by 178 nucleotide polymorphisms (Additional data file 3); a table of STEC O157 genotypes defined by a minimal set of 32 nucleotide polymorphisms (Additional data file 4); a figure showing neighbor-joining tree of polymorphism-derived genotypes tagged with a minimal set of 32 polymorphisms (Additional data file 5).

## Supplementary Material

Additional data file 1 STEC O157 strains used in this study with their corresponding PFGE patterns and polymorphism-derived genotypes. Click here for file

Additional data file 2 Nucleotide polymorphism allele frequencies in STEC O157 strains of bovine and human origin. Click here for file

Additional data file 3 STEC O157 genotypes defined by 178 nucleotide polymorphisms. Click here for file

Additional data file 4 STEC O157 genotypes defined by a minimal set of 32 nucleotide polymorphisms. Click here for file

Additional data file 5 The triplicate sets of numbers on the tree represent bootstrap values from neighbor-joining, parsimony, and maximum-likelihood algorithms, respectively. Asterisks represent bootstrap values below 50%. The outer taxonomic unit genotype numbers correspond with genotype sequences recorded in Additional data file 4. The outer taxonomic units are color coded by genotype for the *tir *255 T>A polymorphism and host origin. The scale bar represents substitutions per site. Click here for file
