# Supplementary figures and images for: Phylogenetic classification of Escherichia coli O157:H7 strains of human and bovine origin using a novel set of nucleotide polymorphisms
Source: Genome Biol. 2009 May 22;10(5):R56. doi: 10.1186/gb-2009-10-5-r56 (PMC2718522; doi:10.1186/gb-2009-10-5-r56)

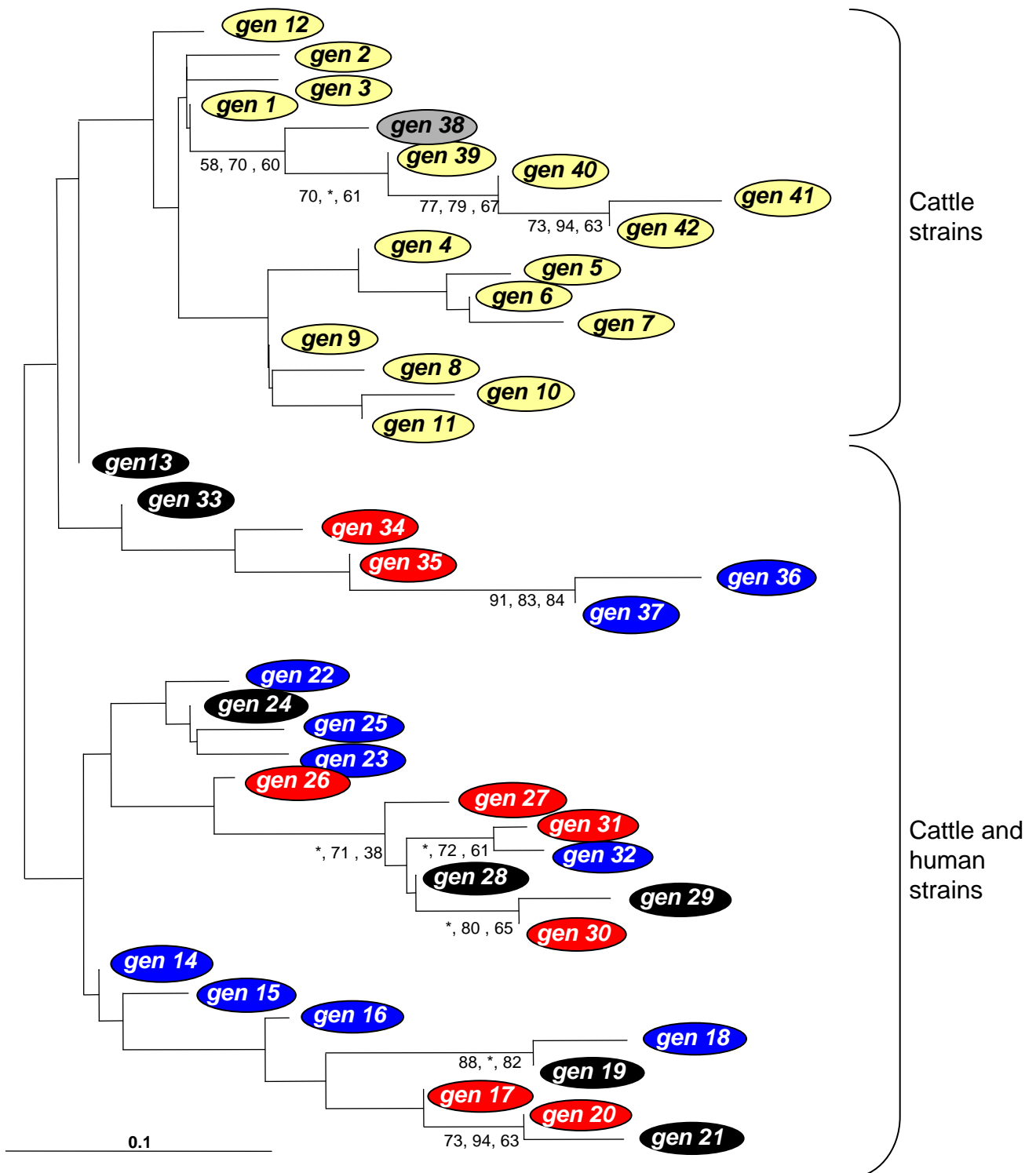

- Cattle *tir* 255 A
- Cattle *tir* 255 A, human *tir* 255 A
- Cattle *tir* 255 T, human *tir* 255 T
- Cattle *tir* 255 T
- Human *tir* 255 T

Supplement: Additional data file 5 — The triplicate sets of numbers on the tree represent bootstrap values from neighbor-joining, parsimony, and maximum-likelihood algorithms, respectively. Asterisks represent bootstrap values below 50%. The outer taxonomic unit genotype numbers correspond with genotype sequences recorded in Additional data file 4. The outer taxonomic units are color coded by genotype for the tir 255 T>A polymorphism and host origin. The scale bar represents substitutions per site. [file gb-2009-10-5-r56-S5.pdf]
